# The effects of treated dried cassava stem replacement on feed intake, digestibility, rumen fermentation, and blood metabolites of Thai native cattle

**DOI:** 10.5713/ab.24.0577

**Published:** 2025-01-24

**Authors:** Sokchea Vong, Thearak Yi, Savdy Net, Sophany Morm, Areerat Lunpha, Chittraporn Yeanpet, Ruangyote Pilajun

**Affiliations:** 1 Department of Animal Science, Faculty of Agriculture, Ubon Ratchathani University, Ubon Ratchathani 34190, Thailand; 2 Department of Animal Science, Faculty of Agriculture and Food Processing, National University of Battambang, Battambang 020202, Cambodia

**Keywords:** Alkaline, Blood Metabolite, Cassava Stem, Digestibility, Rumen Fermentation, Thai Native Cattle

## Abstract

**Objective:**

To investigate the use of cassava stems as an alternative feedstuff for ruminants, a study was conducted measuring the effect of replacing rice straw with untreated and treated dried cassava stems. The study assessed its impact on feed intake, nutrient digestibility, blood metabolites, and the growth performance of Thai native cattle.

**Methods:**

Six male cattle were arranged in a 3×3 replicated Latin square design to receive three treatments. All animals were provided with rice straw *ad libitum* and 14% crude protein (CP) concentrate at 0.5% body weight. Treatment variations consisted of a control group (Ctrl), one group that replaced rice straw with dried cassava stem (DCS), and another group that replaced rice straw with alkali, urea, and Ca(OH)_2_, treated dried cassava stem (tDCS) at 15% of rice straw intake. The experiment spanned three 21-day periods.

**Results:**

Feed intake, body weight gain, and feed conversion ratio (FCR) were similar across treatments. Total tract apparent digestibility for the cattle, which included dry matter, organic matter, CP, neutral detergent fiber, and acid detergent fiber, was higher than those on the tDCS treatment compared to the other groups (p<0.05). Ruminal pH, volatile fatty acid concentration, and blood metabolites remained unaffected by dietary treatments, except for BUN which was increased with tDCS replaced rice straw (p<0.05). Alkali-tDCS helped reduce rectum temperature compared to the control and DCS group (p<0.05).

**Conclusion:**

Replacing rice straw with tDCS improved nutrient digestibility but did not significantly impact feed intake, growth rate, FCR, rumen fermentation, and the blood metabolite of Thai native cattle.

## INTRODUCTION

Cassava (*Manihot esculenta* Crantz) has risen to become one of the most significant food crops globally, surpassed only by rice and maize respectively, due to its crucial role in the human food supply [1]. Cultivating cassava is uncomplicated, as it is tolerant of acidic soils, and can be grown throughout the year via stem cutting [2]. After cultivation, the cassava stem, which makes up about 50% of the root mass, is typically separated from the roots and leaves. While a small portion of the cassava stem is used for planting the next cultivation cycle and some as cooking fuel [3], its low nutritional content limits its use in animal diets. Despite this, it has been shown that the cassava leaf can be a valuable source of protein for ruminants [1].

Cassava stems are often deemed useless for feed due to their notably woody nature. However, the value of using agricultural wastes as ruminant feed can improve with their field abundance. Cassava stems, in both dried and silage forms, have been utilized as ruminant feed, though not extensively [4–6]. Despite significant starch content reports (30% dry matter [DM]) [6], high lignocellulose content can diminish feed intake and digestibility. Nevertheless, an alkaline agent like calcium hydroxide can enhance the nutritive value of agricultural waste by breaking down the cell wall’s ester membrane [7,8]. Urea solution use has been reported to augment the crude protein (CP) content and improve the digestibility of fibrous feed in both *in vitro* [9–11] and *in vivo* [12–14] trials. Furthermore, other alkalines such as NaOH [15,16] and Ca(OH)_2_ or lime [17] have been employed to boost the degradability of fibrous feed for ruminants. Moreover, the urea-lime combination has been tested and found to improve fibrous feed degradability by rumen microbes [18,19]. In a previous study, combination-treated dried cassava stems (DCSs) with a 3.0% urea solution and a 3.0% Ca(OH)_2_ solution showed an increase in the *in vitro* kinetics of gas production and degradability [20]. Feed digestibility has been found to influence heat production in ruminants [21], suggesting that increasing roughage digestibility through alkali treatment might reduce heat stress, as indicated by rectal temperature, thus improving animal performance.

The objective of this study was to examine the impact of substituting part of rice straw with DCS and alkali-treated DCS on the voluntary intake and nutrient utilization in Thai native cattle.

## MATERIALS AND METHODS

### Animal care

The present experiment was reviewed and approved by the Institutional Animal Care and Use Committee of Ubon Ratchathani University (ID#16/2567/IACUC).

### Animal and experimental design

Six male Thai native cattle, aged between 13 to 15 months and with a body weight (BW) of 150±50 kg, were randomly assigned to a 3×3 replicated Latin square design. They were given three different dietary treatments. The control group (T1, Ctrl) was provided with rice straw *ad libitum* and 0.5% of their BW supplemented with concentrate. The other two groups were given either DCS (T2) or treated dried cassava stem ([tDCS] T3), each accounting for 15% of the control’s rice straw intake on a DM basis. T2 and T3 were chosen based on *in vitro* trials, where they showed the highest degradability and kinetic gas production [20]. The experiment consisted of three 21-day periods, resulting in total assessment duration of 63 days.

### Feed preparation

Bands of rice straw (*Oryza sativa* L. ssp. indica cv. KDML 105) were purchased from a local market to serve as a roughage source for cattle. The concentrate was formulated using common feedstuffs, with cassava chips and soybean meal acting as chief sources of starch and protein respectively. Cassava stems (*Manihot esculenta* Crantz cv. KU50) were sourced from a farmer’s field in the Warin Chamrap district, Ubon Ratchathani, Thailand. The stem, excluding the green top (petioles, stem, and leaf), was cut 10 cm over the root, chopped into pieces less than 1 cm by machine, then sun-dried for three consecutive days. After verifying that the moisture level was below 15%, the stems were stored in plastic bags. The tDCS was prepared following a process outlined in a previous study [20], involving the dissolution of 3.0% urea, 2.0% calcium hydroxide, and 3.0% molasses in tap water, which was then mixed with DCS at a 1:1 ratio. These tDCS were kept in a 200-liter plastic bucket for 21 days before being used for cattle feed. The feed ingredients and chemical compositions of the experimental diets are detailed in Table 1.

### Data and sample collection

Cattle were kept in separate pens with freely access to fresh water and mineral blocks. Ahead of the first period, the cattle were acclimated to the experimental conditions; this involved feeding them for seven days and administering Ivermectin and vitamin AD_3_E injections. The cattle were fed twice daily, at 8:00 AM and 4 PM. Rice straw was provided *ad libitum*, whereas the concentrate was supplemented at 0.5% BW. DCS and tDCS were given to groups T2 and T3 respectively, making up 15% of the control group’s rice straw intake from the previous day by thoroughly blending into the concentrate with each meal. Every day, the quantity of feed offered and refused includes the rice straw, concentrate, and either DCS or tDCS - concentrate mixture was recorded. The cattle were weighed at the start and on the last day of each experimental period to modify feeding concentrates to 0.5% BW. The average daily gain (ADG) was calculated from the difference between the initial and final BW divided by the number of days within each period. Similarly, the feed conversion ratio (FCR) was calculated as the total DM intake (DMI) divided by BW gain.

Feed and ort samples were taken weekly and dried in a 70°C oven. Fecal samples were collected daily from individual cattle via rectal grab sampling during the final five days of each period and were then stored in a −20°C freezer. Rumen fluids were extracted before and 4 h post-morning feeding on the last day of each period using a stomach tube connected to a vacuum pump. The pH of these fluids was immediately measured with a pH meter (HI-83141-1; RS Pro, Bremen, Germany). Forty-five ml of rumen fluids were fixed with 5 mL of 1M H_2_SO_4_ and stored in a 4°C refrigerator. A total of 10 mL blood samples were collected from a jugular vein with a vacuum blood collection tube, and a digital thermometer detected the rectal temperature of each cattle (RS Pro).

### Laboratory analysis

Feed and ort samples were ground through a 0.5 mm screen and analyzed on DM, total ash, CP, and acid insoluble ash (AIA) according to AOAC [22], while neutral detergent fiber (NDF) and acid detergent fiber (ADF) according to Van Soest et al [23]. After thawing the frozen feces samples, 72 hours 60°C oven-dried samples were ground and analyzed as feed samples. The AIA proportion in feed, ort, and feces indicated an estimation of the total tract apparent digestibility according to Van Keulen and Young [24].

The rumen fluid sample was mixed with 1M H_2_SO_4_ and then centrifuged at 2,054×g for 15 min; after which, the supernatant was used to analyze the volatile fatty acid (VFA) concentration. The supernatant was filtered using a 0.45-μm Millipore filter before injection into the chromatographic apparatus. An Ultimate 3000 HPLC (Thermo Fisher Scientific, Waltham, MA, USA), coupled with a C18 (4.6×250 mm) column (Chromaleon Dionex Corp) and UV-Vis detection at 210 nm, was utilized. The mobile phase was 0.005 mol/L H_2_SO_4_ [25].

Blood samples were sent to the U Wellness Center Co. Ltd., in Thailand, for glucose, creatinine, triglyceride, total protein, and blood urea nitrogen (BUN) analyses. The plasma glucose was examined using the glucose oxidase method, while creatinine was analyzed via the isotope dilution mass spectrometry (IDMS) traceable method. The glycerophosphate O method was employed for analyzing the triglyceride. Analysis for the total protein (serum) was conducted through the biuret lithium method, and the urease method was used for BUN analysis [26,27].

### Statistical analysis

All data from the experiment were statistically analyzed using the GLM procedure of SAS OnDemand (SAS Institute Inc., USA). The following model of replicated Latin square design was used:


$${\text{Y}}_{\text{hijk}}=\mu +{\text{S}}_{\text{h}}+{\text{M}}_{\text{i}}+{\text{A}}_{\text{j}}+{\text{P}}_{\text{k}}+{ɛ}_{\text{ijk}}$$

where

Y_hijk_ = observation from beef j, receiving diet I, during period k
μ = the overall mean
S_h_ = replicated square
M_i_ = the effects of the treatment i
A_j_ = the effect on the animal j
P_k_ = the effect of period k
ɛijk residual effects

The results were presented as mean values, accompanied by the standard error of the mean. The differences between treatment means were determined by the least significant difference, with a 95% confidence interval. Significant differences were defined by a value of p<0.05.

## RESULTS

### Feed intake and growth performance

The intake of rice straw was lower in both the DCS and tDCS groups compared to the control group (p<0.05). However, there was no significant difference in the intake of total roughage and concentrate, measured in both units of kg/d, %BW, and g/kg BW^0.75^, between the groups (p>0.05). The total intake (kg/d) of the control group did, however, tend to be slightly higher than the other treatment groups (p<0.10). The BW gain and ADG were similar among the control, DCS, and tDCS groups. Therefore, FCR showed no difference between the cattle groups (p>0.05, Table 2).

### Nutrient digestibility

Replacing rice straw with tDCS at 15% of rice straw intake in Thai native cattle demonstrated higher apparent digestibility of DM, organic matter (OM), CP, NDF, and ADF compared to the control (p<0.05, Table 3). Cattle fed with DCS exhibited a higher digestibility of DM and NDF than the control (p<0.05), but not OM or ADF. However, the digestibility of CP was lower in cattle fed with DCS than in other groups (p<0.05).

### Ruminal pH and volatile fatty acid concentration

The pH of rumen fluid and the concentrations of acetic acid (C2), propionic acid (C3), and the ratio of C2 to C3 at 0 and 4 h after the morning feeding of cattle were not significantly different between treatments (p>0.05). The proportion of butyric acid (C4) in rumen fluid 4 h after the morning meal in the control group was higher than in cattle fed with DCS (p<0.05), but there was no significant difference when compared to cattle fed with tDCS. Furthermore, before morning feeding, the mean proportion of C4 in rumen fluid was similar among all cattle groups (Table 4).

### Rectal temperature and blood metabolite

The mean rectal temperature at 4 h post-morning feeding was significantly different between cattle groups (p<0.05), where tDCS was lower than the control and DCS (Table 5). Blood metabolites of Thai native cattle, such as glucose, creatinine, triglyceride, and total protein, were unaffected by DCS or tDCS replacement (p>0.05), except for BUN. Rice straws substituted with tDCS demonstrated higher BUN than those replaced with DCS or left un-replaced (p<0.05).

## DISCUSSION

### Chemical composition of experimental diets

It was demonstrated that the CP of tDCS increased with urea, calcium hydroxide, and molasses-treated samples. Lower NDF and ADF were found in tDCS compared to DCS, a finding that aligns with Thuy Hang and Preston [3] who noted that treating freshly chopped cassava stem with varying levels of urea (0% to 5% DM), could linearly decrease NDF and ADF contents. Additionally, treatments using urea and/or alkali substances like NaOH [16] and Ca(OH)_2_ [28] have been shown to reduce the fiber fraction of straw. This reduction in NDF and ADF proportions in fibrous feed may be attributed to the breakdown of lignocellulose due to alkali properties [7]. However, a past study Vong et al [20] reported that treatment with 3% urea and 2% Ca(OH)_2_ did not alter the fiber proportion of DCS. This may be due to the inherent properties of cassava stem harvested from the field, such as the growth stage, soil fertility, and other cultivation factors.

### Feed intake and growth performance

The cattle successfully consumed DCS or tDCS mixed with concentrate. This was possibly due to its soft part, ground to less than 1 cm, and treated with an alkali solution, improving palatability. Replacement of rice straw with DCS or tDCS did not influence total intake, growth, or the cattle’s feed utilization efficiency. This may result from replacing the rice straw intake with tDCS, which is a similar source of fiber. Such findings concur with Sengtin [4], who reported total intake and BW change were consistent between milking cows fed on a total mixed ration containing DCS or fermented cassava stem. However, numerous studies indicate that cattle’s intake of fibrous feed increases with alkaline treatments [12,19], though some do not [14,29]. Such differences may result from the roughage type that contains varying proportions of NDF, ADF, and lignin, as well as the structure of lignocellulose. The current study discovered that cassava stems contain 19.2% hemicellulose. In contrast, Sengtin [4] recorded 52.2% hemicelluloses, 12.0% cellulose, and 15.1% lignin; and rice straw contains 38.4% hemicellulose, 40.5% cellulose, and 6.27% lignin [16]. Additionally, sugarcane bagasse contains different hemicellulose (5.51% DM), cellulose (54.6% DM), and lignin (14.3% DM) proportions compared to cassava stems and rice straw [30]. This could be due to variations in the crop harvesting period. Similarly, differences in the types and doses of alkaline-treated conditions resulted in fiber structure differences, impacting ruminant intake. Roughage intake was elevated when sugarcane bagasse was treated with 4% urea at a 66% moisture level in beef cattle [12]; contrastingly, 4.0% urea-treated sugarcane bagasse with 40% moisture did not affect the intake of crossbred bulls [31]. Likewise, the BW changes and feed conversion rates in this study’s cattle groups could relate to the lack of difference within roughage and concentrate intakes. Stehr et al [32] reported that beef cattle fed 5.0% CaO-treated barley straw had similar feed intake, ADG, and feed efficiency compared to cattle fed untreated barley straw or corn silage. Hence, combining the pretreatment of fibrous feed with alkali under suitable conditions may be an essential area for further research to increase feed intake and enhance ruminants’ production performance.

### Nutrient digestibility

Improvements in nutrient digestibility due to alkaline-treated tDCS replacement in cattle may stem from the disruption of interpolymer bonds among cellulose, pectin, and hemicellulose in the cell wall, which allows for easier degradation by rumen microorganisms. Qingxiang [33] argued that to enhance the digestibility of crop residues, a key method is a chemical treatment aimed at removing encrusting substances like cellulose, hemicellulose, and lignin, using NaOH, ammonia (NH_3_), and Ca(OH)_2_. Additionally, treating straw with NaOH or feed-grade urea (ammonia) frees up fermentable cell wall components from lignin-associated bonds, enhancing their rumen fermentation [8]. This study’s results align with Polyorach and Wanapat [18] and Khejornsart and Jantanam [19], who reported that rice straw treatments using urea or urea+Ca(OH)_2_ significantly improved digestibility in beef cattle and swamp buffaloes, respectively. Furthermore, the diet of lactating Holstein cows showed significantly higher OM and NDF digestibility when supplemented with NaOH/ethanol-H_2_O-treated corn stover ration when compared to diets augmented with untreated stover, although DM digestibility did not increase [7]. Contrarily, a prior study [20] indicated that the *in vitro* degradability of DCS did not increase with treatment using 2.0% to 3.0% urea or Ca(OH)_2_, or their combination. Stehr et al [32] suggested that treating barley straw with 5.0% CaO had no significant effect on nutrient digestibility in feedlot cattle. Sengtin [4] also discovered that the digestion coefficients of dairy cows were unaffected by the roughage sources in a total mixed ration that included either fermented cassava stem or dry cassava stem.

These variations in findings may stem from differing conditions in each experiment, including the type of fibrous feedstuffs, the kind and dosage of alkaline used, other experimental diets, and the species of ruminant plus their physiological stage.

The higher NDF and ADF digestibility in cattle fed DCS or tDCS, compared to the control, could be attributed to the superior digestibility of fiber from DCS over rice straw. As shown in Table 1, the fiber fractions found in rice straw appear to be greater than those in DCS and tDCS. The similar feed intake across treatments indicates that the control group experienced higher fiber intake than those fed DCS and tDCS. This is consistent with studies that suggest nutrient digestibility decreases as NDF levels increase, as observed in Black Angus×Zebu crossbred beef cattle [34] and Lezhi black goats [35]. A cassava stem can be divided into two parts: the outer and inner elements. The external portion consists of hard fibers, while the internal section contains soft fibers. Martín et al [36] reported that 35% to 67% of glucans found in cassava stems accounted for 18.5% to 42.4% of starch. This finding aligns with Idris et al [37], who found 43.0% of carbohydrates in cassava stems. These starch or carbohydrate fractions, believed to come from the inside portion of the cassava stem, are easily degraded by rumen microbes. Furthermore, while nutrient digestibility was increased, total intake remained unchanged, possibly related to the capacity limit of the rumen. This is supported by Donnelly et al [7], who noted that while the DM and NDF digestibility of NaOH/ethanol-H_2_O treated corn stover improved to levels akin to soy hulls, neither total DM nor NDF intakes changed in milking cows. Despite the potential for high digestibility to encourage greater consumption, daily intake may be constrained by gut fill [32].

### Ruminal pH and volatile fatty acid concentration

Though nutrient digestibility varied significantly among treatments, ruminal pH and fermentation end-products, such as VFA concentration, were consistent. This consistency could be due to VFA absorption through the rumen wall and utilization by ruminant body tissue. It might also be attributed to the low supplementation of concentrate, at 0.5% of BW, which led to a lower VFA concentration in the rumen. Most of the VFA is then absorbed into the blood system. Khejornsart et al [38] determined that VFA concentration in the rumen was unaffected despite swamp buffalo consuming urea-lime-treated rice straw instead of untreated straw. Similarly, the concentration of VFA in the rumen did not vary based on the type of roughage (un-fermented vs. 3.0% urea-fermented sugarcane bagasse) in sheep consuming a 2.0% BW concentrate [14]. There have, however, been reports of increased C3 concentration associated with digestibility in cattle fed 4.0% urea or 2.0% urea+2.0% Ca(OH)_2_-treated, compared to untreated sugarcane bagasse [12] and in swamp buffalo fed 2.0% or 2.0% urea+2.0% lime-treated rice straw [19]. Ruminal VFA absorption is linked with the concentration gradient of individual VFA between ruminal fluid and epithelial blood [39]. Therefore, a low nutrient input leading to a low VFA concentration may explain the lack of effect from dietary treatments. Moreover, the reduced C4 proportion at 4 h of feeding in cattle consuming DCS rather than the control might be linked to the C3 proportion, though it was not significantly different among treatments. The high starch content (~30% DM) of the cassava stem [6], could mean that the digestion of DCS by rumen microbes generates more C3, thereby lowering the C4 proportion compared to the control group fed solely on rice straw.

### Rectal temperature and blood metabolite

The rectal temperature of cattle decreased when fed with tDCS, dropping to within the standard range for *Bos indicus* cattle, which is between 39.0°C and 39.3°C, as reported by Moura et al [40]. A cow’s body temperature can vary based on numerous factors, such as ambient temperature, humidity, and metabolic changes tied to physiological state and digestion. Under conditions of heat stress, Rejeb et al [41] found that dairy cows’ rectal temperature had a positive correlation with digestibility (r = 0.64), but a strong negative correlation with DMI (r = −0.85) and milk yield (r = 0.89). However, the rectal temperature did not change when the cows were fed a progressively fermented matter-rich diet [42]. Therefore, the fluctuations in rectal temperatures noted in our study may not be attributable to digestibility, despite the reported negative correlation between heat production and CP digestibility [21], but could be due to other factors. Typically, cattle’s body temperature decreases with water consumption, a variable directly affected by DMI and feed characteristics [43].

BUN increased in cattle receiving tDCS supplementation as a substitute for rice straw intake. This may be attributed to the 2.0% urea used to treat DCS as an alkaline source. This finding is consistent with the study by Chanjula et al [44], who also observed elevated BUN in Thai native-Anglo Nubian goats fed with ensiled oil palm frond containing 5.0% urea or 2.5% urea+2.5% Ca(OH)_2_. Similarly, Wanapat et al [45] reported that lactating dairy cows exhibited higher BUN when consuming 5.5% urea or 2.2% urea+2.2% Ca(OH)_2_-treated rice straw compared to a control group. The availability of ammonia nitrogen in the rumen, primarily derived from feed, is a key factor influencing the urea level in ruminants’ bloodstreams [46]. Other considerations include the liver’s nitrogen recycling capacity and nitrogen utilization by rumen microbes [47]. However, BUN remained unchanged in Thai native sheep fed either fermented sugarcane bagasse without additives or bagasse with 10% molasses and 3.0% urea [14]. Similarly, no BUN variance was noted when beef cattle were fed 4.0% urea-treated-, 2.0% urea+2.0% lime-treated-, or untreated sugarcane bagasse [12]. These varying effects could be linked to factors such as each ruminant type’s nitrogen recycling capacity [48], rumen microorganism fermentation efficiency, and the experimental diets used. Since BUN levels act as a crucial performance indicator of nitrogen and carbohydrate synchronization in the rumen, high BUN may imply that excess nitrogen is not involved in microbial protein synthesis due to inadequate synchronization.

## CONCLUSION

Although the feed intake, rumen fermentation, and blood metabolites of Thai native cattle were not significantly altered by DCS or tDCS replacement, combining urea and Ca(OH)_2_ as an alkaline solution to treat the DCS improved the nutritive value of this crop residue, leading to increased digestibility. DCS is an under-utilized crop residue on the farm. Therefore, further investigation should be conducted on feeding DCS or alkaline-treated DCS in consortium with a high concentrate proportion to produce ruminants, as this could promote productive efficiency and reduce feed costs.

## Figures and Tables

**Table 1:** Ingredients and chemical composition of the dietary treatment used in the experiment

Items	Concentrate	RS	DCS	tDCS
Ingredient (% DM)				
Cassava chip	30			
Corn meal	20			
Rice bran	14			
Palm kernel meal	10			
Soybean meal	23			
Molasses	1.0			
Palm oil	0.5			
Premix	0.5			
Dicalcium phosphate	0.5			
Salt	0.5			
Chemical composition (% DM)				
Dry matter	91.3	91.5	93.1	95.8
Organic matter	92.8	88.0	90.8	93.3
Crude protein	17.7	3.76	7.39	11.0
Neutral detergent fiber	23.3	74.9	65.1	42.7
Acid detergent fiber	14.7	47.2	45.9	38.8

RS, rice straw; DCS, dried cassava stem; tDCS, dried cassava stem treated with 3% urea, 3% molasses, and 2% Ca(OH)_2_; DM, dry matter.

**Table 2:** The effect of replacing rice straw with dried cassava stem and treated dried cassava stem on growth performance in Thai native cattle

Items	Ctrl	DCS	tDCS	SEM	p-value
Rice straw intake (kg DM/d)	1.89^a^	1.55^b^	1.59^b^	0.136	<0.001
DCS or tDCS intake (kg DM/d)	-	0.28	0.28	-	-
Total roughage intake (kg DM/d)	1.89	1.83	1.88	0.007	0.1850
%BW	1.07	1.09	1.03	0.005	0.3216
g/kg BW^0.75^	54.1	54.2	52.3	0.240	0.3496
Concentrate intake (kg DM/d)	1.11	1.03	1.09	0.006	0.1113
%BW	0.63	0.61	0.54	0.004	0.4207
g/kg BW^0.75^	31.5	30.4	27.5	0.189	0.3594
Total intake (kg DM/d)	2.99	2.87	2.88	0.010	0.0843
%BW	1.70	1.69	1.56	0.007	0.3707
g/kg BW^0.75^	85.5	84.7	79.8	0.336	0.3607
Body weight change (kg)	7.34	9.31	11.2	0.135	0.2048
Average daily gain (kg/d)	0.37	0.44	0.53	0.005	0.1932
Feed conversion rate	9.62	8.56	6.64	0.162	0.4907

Ctrl, control; DCS, dried cassava stem; tDCS, dried cassava stem treated with 3% urea, 3% molasses, and 2% Ca(OH)_2_; DM, dry matter; BW, body weight; SEM, standard error of means.

a,b Mean on the same row with different superscripts are significant different (p<0.05).

**Table 3:** The effect of replacing rice straw with dried cassava stem and treated dried cassava stem on apparent nutrient digestibility in Thai native cattle

Nutrient digestibility (%)	Ctrl	DCS	tDCS	SEM	p-value
Dry matter	54.4^c^	57.3^b^	62.1^a^	0.141	0.0079
Organic matter	56.9^b^	59.9^b^	65.1^a^	0.157	0.0100
Crude protein	52.1^b^	46.5^c^	63.5^a^	0.150	0.0003
Neutral detergent fiber	42.4^c^	54.9^b^	62.1^a^	0.145	<0.0001
Acid detergent fiber	46.7^b^	51.5^b^	63.0^a^	0.219	0.0039

Ctrl, control; DCS, dried cassava stem; tDCS, dried cassava stem treated with 3% urea, 3% molasses, and 2% Ca(OH)_2_; DM, dry matter; BW, body weight; SEM, standard error of means.

a–c Mean on the same row with different superscripts are significant different (p<0.05).

**Table 4:** Effect of replacing rice straw with dried cassava stem and treated dried cassava stem on rumen fermentation in Thai native cattle

Items	Ctrl	DCS	tDCS	SEM	p-value
Ruminal pH					
0 h post feeding	7.3	7.5	7.6	0.016	0.3390
4 h after feeding	6.9	6.9	7.1	0.015	0.4876
Mean	7.1	7.3	7.2	0.014	0.4022
Total volatile fatty acid (TVFA) (mM/L)					
0 h post feeding	86.6	79.9	80.6	0.839	0.2348
4 h after feeding	106	115	91.0	1.586	0.2761
Mean	96.1	97.3	85.8	1.014	0.3362
Acetic acid (% TVFA)					
0 h post feeding	50.4	52.2	51.6	0.694	0.3932
4 h after feeding	55.6	54.7	54.3	0.736	0.3535
Mean	53.0	53.5	53.0	0.532	0.2540
Propionic acid (% TVFA)					
0 h post feeding	37.7	36.8	36.4	0.875	0.4266
4 h after feeding	27.4	33.3	32.9	0.803	0.3650
Mean	32.6	35.1	39.7	0.729	0.1485
Butyric acid (% TVFA)					
0 h post feeding	11.9	11.0	12.0	0.190	0.5732
4 h after feeding	17.0^a^	12.0^b^	12.8^ab^	0.231	0.0141
Mean	14.5	11.5	12.4	0.195	0.3706
Acetic acid : Propionic acid					
0 h post feeding	1.73	2.01	1.85	0.060	0.3074
4 h after feeding	3.08	2.93	2.82	0.083	0.3072
Mean	2.91	2.77	3.32	0.065	0.5488

Ctrl, control; DCS, dried cassava stem; tDCS, dried cassava stem treated with 3% urea, 3% molasses, and 2% Ca(OH)_2_; SEM, standard error of means.

a,b Mean on the same row with different superscripts are significantly different (p<0.05).

**Table 5:** Effect of replacing rice straw with dried cassava stem and treated dried cassava stem on rectal temperature and blood metabolite in Thai native cattle

Items	Ctrl	DCS	tDCS	SEM	p-value
Rectal temperature (°C)					
0 h post feeding	38.7	38.8	38.7	0.013	0.1660
4 h after feeding	39.5^a^	39.6^a^	39.2^b^	0.014	0.0125
Mean	39.1^ab^	39.2^a^	38.9^b^	0.011	0.0042
Blood metabolite					
Glucose (mg/dL)	73.0	82.3	78.3	0.508	0.0929
Creatinine (mg/dL)	1.52	1.45	1.43	0.005	0.3177
Triglyceride (mg/dL)	38.3	34.3	36.2	0.457	0.1970
Total protein (g/dL)	6.35	6.37	6.25	0.015	0.0925
Blood urea nitrogen (mg/dL)	9.67^b^	10.8^b^	12.8^a^	0.086	0.0065

Ctrl, control; DCS, dried cassava stem; tDCS, dried cassava stem treated with 3% urea, 3% molasses, and 2% calcium hydroxide; SEM, standard error of means.

a,b Mean on the same row with different superscripts are significantly different (p<0.05).
